# Wheat field earthworms under divergent farming systems across a European climate gradient

**DOI:** 10.1002/eap.3066

**Published:** 2024-11-25

**Authors:** Visa Nuutinen, Maria J. I. Briones, Stefan Schrader, Igor Dekemati, Nikola Grujić, Juha Hyvönen, Mari Ivask, Simon Bo Lassen, Eva Lloret, Irene Ollio, Paula Pérez‐Rodríguez, Barbara Simon, Merit Sutri, Nancy de Sutter, Kristian K. Brandt, Krista Peltoniemi, Merrit Shanskiy, Lieven Waeyenberge, Silvia Martínez‐Martínez, David Fernández‐Calviño

**Affiliations:** ^1^ Soil Ecosystems Natural Resources Institute Finland (Luke) Jokioinen Finland; ^2^ Departamento de Ecología y Biología Animal Universidade de Vigo Vigo Spain; ^3^ Institute of Biodiversity, Thünen Institute Braunschweig Germany; ^4^ Department of Agronomy Institute of Crop Production Sciences, Hungarian University of Agriculture and Life Sciences Gödöllö Hungary; ^5^ Department for Entomology and Agricultural Zoology Institute for Phytomedicine, Faculty of Agriculture, University of Belgrade Belgrade‐Zemun Serbia; ^6^ Applied Statistical Methods, Natural Resources Institute Finland (Luke) Rovaniemi Finland; ^7^ Institute of Agricultural and Environmental Sciences, Estonian University of Life Sciences Tartu Estonia; ^8^ Department of Plant and Environmental Sciences University of Copenhagen Frederiksberg C Denmark; ^9^ Sustainable Use, Management and Reclamation of Soil and Water Research Group (GARSA), Department of Agricultural Engineering Universidad Politécnica de Cartagena Cartagena Spain; ^10^ Section for Soil Science and Agricultural Chemistry, Department of Plant Biology and Soil Science Universidade de Vigo Ourense Spain; ^11^ Department of Soil Science Institute of Environmental Sciences, Hungarian University of Agriculture and Life Sciences Gödöllö Hungary; ^12^ ILVO (Flanders Research Institute for Agriculture, Fisheries and Food), Plant Sciences Unit Merelbeke Belgium; ^13^ Soil Ecosystems, Natural Resources Institute Finland (Luke) Helsinki Finland

**Keywords:** arable fields, climate change, farming systems, global warming, macrofauna, organic farming, regional distributions, soil biodiversity

## Abstract

Earthworms are a key faunal group in agricultural soils, but little is known on how farming systems affect their communities across wide climatic gradients and how farming system choice might mediate earthworms' exposure to climate conditions. Here, we studied arable soil earthworm communities on wheat fields across a European climatic gradient, covering nine pedo‐climatic zones, from Mediterranean to Boreal (S to N) and from Lusitanian to Pannonian (W to E). In each zone, 20–25 wheat fields under conventional or organic farming were sampled. Community metrics (total abundance, fresh mass, and species richness and composition) were combined with data on climate conditions, soil properties, and field management and analyzed with mixed models. There were no statistically discernible differences between organic and conventional farming for any of the community metrics. The effects of refined arable management factors were also not detected, except for an elevated proportion of subsurface‐feeding earthworms when crop residues were incorporated. Soil properties were not significantly associated with earthworm community variations, which in the case of soil texture was likely due to low variation in the data. Pedo‐climatic zone was an overridingly important factor in explaining the variation in community metrics. The Boreal zone had the highest mean total abundance (179 individuals m^−2^) and fresh mass (86 g m^−2^) of earthworms while the southernmost Mediterranean zones had the lowest metrics (<1 individual m^−2^ and <1 g m^−2^). Within each field, species richness was low across the zones, with the highest values being recorded at the Nemoral and North Atlantic zones (mean of 2–3 species per field) and declining from there toward north and south. No litter‐dwelling species were found in the southernmost, Mediterranean zones. These regional trends were discernibly related to climate, with the community metrics declining with the increasing mean annual temperature. The current continent‐wide warming of Europe and related increase of severe and rapid onsetting droughts will likely deteriorate the living conditions of earthworms, particularly in southern Europe. The lack of interaction between the pedo‐climatic zone and the farming system in our data for any of the earthworm community metrics may indicate limited opportunities for alleviating the negative effects of a warming climate in cereal field soils of Europe.

## INTRODUCTION

Earthworms contribute to soil processes and fertility in many ways: by breaking down decaying plant residues and moderating nutrient cycles, by biological regulation when grazing on microorganisms, and, as ecosystem engineers, by improving soil structure and providing habitats for other soil organisms (Blouin et al., 2013; Turbé et al., 2010; Vidal et al., 2023). In arable soils, earthworms are important as they enhance soil fertility. Two research syntheses have summarized the positive effects of earthworms on plant growth and yield (Scheu, 2003; van Groenigen et al., 2014) while a recent global analysis estimated that in European cereal cultivation, earthworms increase yields by approximately 7%–8% and the absolute production by close to 40 million metric tons (Fonte et al., 2023). This occurs importantly through increased nitrogen availability due to accelerated decomposition and nutrient release (van Groenigen et al., 2014) and improved soil structure (Andriuzzi et al., 2015), but also through the enhancement of plant growth‐promoting microbes and reduction of potential plant pathogens (Hodson et al., 2023).

The abundance of earthworms varies widely in agricultural soils. In a global synthesis, the mean abundance in herbaceous croplands was 50 individuals m^−2^ (Phillips et al., 2019). That value is toward the low end of variation for European agricultural soils, with a maximal abundance of approximately 300 individuals m^−2^ in row‐cropped agricultural soils and more than 600 individuals m^−2^ in pastures (Edwards & Arancon, 2022). In cultivated mineral soils, inherent soil properties set the general frame for the variation in earthworm abundance, with soil texture being one of the main controlling factors, and from this, medium coarse textures seem to be the most favorable for earthworms (Guild, 1948; Nieminen et al., 2011).

Contrary to natural environments, arable systems such as cereal fields typically persist in an early stage of succession due to seasonal sequence of seedbed preparation, seeding, harvest, and tillage. This often impedes the development of species‐rich earthworm communities, and within each field, the richness (alpha diversity) of earthworms is typically low, the global mean being estimated at two species in herbaceous production fields (Phillips et al., 2019). Low richness can be nevertheless meaningful for soil properties if it consists of functionally different species. Depending on their morphological and ecological characteristics, three main groups of earthworms can be distinguished (Bottinelli & Capowiez, 2021; Bouché, 1977): epigeic litter dwellers, endogeic mineral‐soil burrowers, and anecic vertical burrowers. Epigeics and anecics are primary decomposers, which feed on energy‐rich organic material deposited on the soil surface, whereas endogeics as secondary decomposers feed on more energy‐poor, highly processed belowground organic material.

In field crop cultivations, earthworms are affected by the frequency and intensity of tillage (Briones & Schmidt, 2017; van Cappelle et al., 2012), quality of chemical inputs (both fertilizers; Curry, 2004 and pesticides; Pelosi et al., 2013, 2014), the type of crop and the number of different crops in rotation (Torppa & Taylor, 2022), application of cover‐ and intercrops (Roarty et al., 2017; Schmidt et al., 2003), and crop residue management (Melman et al., 2019). Low‐input management is often advantageous for earthworms, reduction of tillage depth, and intensity being an example of a generally enhancing factor, which may also impact specific ecological types (Briones & Schmidt, 2017).

Geographical variation of earthworm communities is wide and climatic factors such as yearly precipitation and mean temperature are major drivers of abundance, species composition, and geographical distribution (Phillips et al., 2019). The variation of earthworm abundance and richness in Europe points to a strong climatic impact. Abundance and species richness are both high in Atlantic zones, which receive relatively high rainfall, and are much lower in the warmer and drier Mediterranean zones (Phillips et al., 2019; Rutgers et al., 2016). The impacts of drought on earthworms have not been studied extensively (Singh et al., 2019), but findings from field experimental (Briones et al., 2009) and observational studies (Eggleton et al., 2009) indicate negative effects on their diversity and abundance. A major environmental challenge currently facing Europe is extreme weather, with unprecedentedly high summer temperatures and related severe droughts, particularly in the Mediterranean zones (van der Wiel et al., 2023; Vicente‐Serrano et al., 2014). This will have direct and indirect, mainly negative consequences for earthworm communities (Hiltpold et al., 2017; Singh et al., 2019).

Harnessing the soil biota community is essential in organic farming where plant nutrition is based on the efficient functioning of the soil decomposer web fueled by organic fertilizers (Bertrand et al., 2015). A research synthesis by Bengtsson et al. (2005) suggests that in the case of earthworms, the engagement is often successful as their abundance under organic farming was higher than in conventional management. Similar findings were reported in a subsequent synthesis by Christel et al. (2021). Geographically broad studies with systematic focus on contrasting agricultural systems' effects on earthworm communities remain rare (but see Tsiafouli et al., 2015). In this study, we therefore investigated the variation of earthworm communities in conventional and organic cereal cultivation under a wide range of European climatic conditions. We also elucidated the relationship of earthworm community structure with local soil conditions and refined the properties of field management. Studying the variation of communities across a large latitudinal climate gradient is essential to understand the potential consequences of climate change on soil biodiversity and soil functioning (De Frenne et al., 2013). It can also help address the topical question of whether the potential negative effects of climate change could be alleviated by widespread adoption of any particular sustainable land management over another (de Vries et al., 2012; Droste et al., 2020).

The specific research questions were: (1) How do wheat field earthworm communities vary across European pedo‐climatic zones and what are the main environmental drivers of their variation? (2) Do communities in organically and conventionally farmed systems differ, and is there evidence for interaction between farming system and environmental conditions? (3) How does the variation of field management practices within the farming systems, such as tillage, fertilization, crop rotation, and use of pesticides, affect community metrics?

## MATERIALS AND METHODS

### Field sites

**FIGURE 1:**
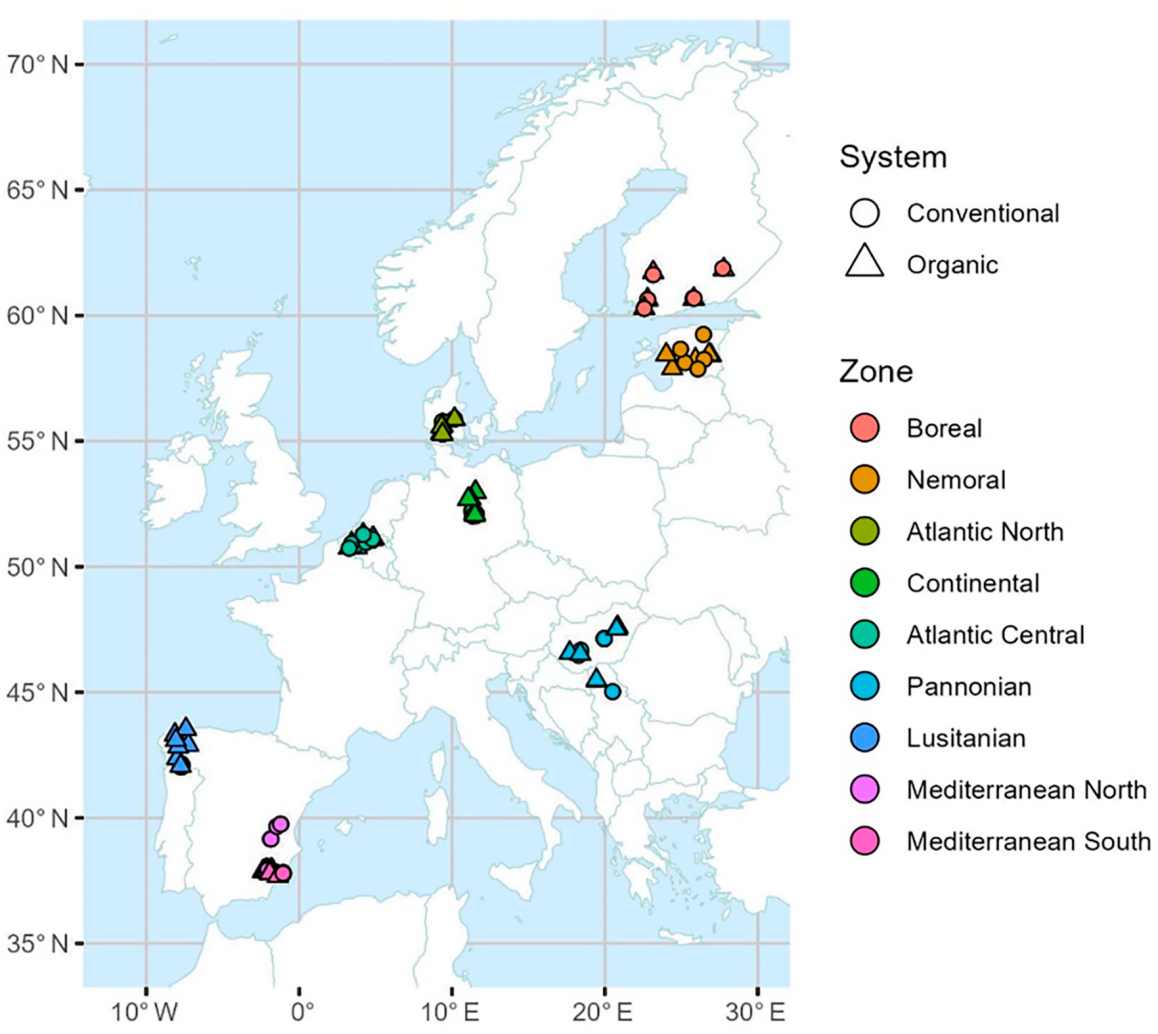
The field site locations at the nine European pedo‐climatic zones. Not all sites are visible due to overlying symbols. For the number of fields in each zone, see Appendix S1: Table S1.

The study covered nine pedo‐climatic zones in Europe (Figure 1; EU, 2011). From north to south, the zones and representative countries were Boreal (Finland), Nemoral (Estonia), Atlantic North (Denmark), Atlantic Central (Belgium), Continental (Germany), Pannonian (Hungary and Serbia), Lusitanian (northwestern Spain), and Mediterranean South and North (low and high elevation areas in southern and more northern Spain, respectively). Within each zone, 10 wheat fields under long‐term conventional or organic farming were selected for the study (for the few exceptions with higher number of fields, see Appendix S1: Table S1). When possible, two fields from an organic and a conventional farm situated in the same locality were selected. In many cases, it was, however, not possible to form such distinct farm pairs, and fields from separate localities were included. The organically managed fields fulfilled EU criteria of organic production (EU, 2018) except for two fields that had been in organic management for less than two years and were still in transition phase to organic farming. Of the remaining 93 organic fields, 65 had been in organic farming for more than 10 years and 28 fields for 3–10 years.

### Earthworm sampling and processing

Efficient earthworm sampling requires that earthworms are active in the topsoil, and at each pedo‐climatic zone, the single sampling matched up as far as possible with such conditions. In most of the zones, fieldwork took place in 2019, but exceptionally severe drought conditions postponed the sampling at the two Mediterranean zones and Pannonian zone until 2020 (Appendix S1: Table S1). Samples were taken in post‐harvest conditions prior to any physical disturbance of the soil to guarantee comparable physical conditions during sampling.

At each field, earthworm samples were taken within a representative area of 1 ha delimited for the fieldwork. Three quadrats (0.5 m × 0.5 m) were positioned at the apexes of an equilateral (5 m) triangle. In Boreal and Mediterranean zones, an area of 0.25 m × 0.25 m was used due to local soil conditions, which made the hand‐sorting exceptionally arduous. Sampling depth was 0.25 m.

At the place of sampling, the quadrat was placed on the soil surface, and any vegetation present was cut and removed together with the surface litter after it had been checked for the presence of earthworms. A soil sample was then excavated using a spade, removing maximally large intact blocks to avoid damaging the specimens. Earthworms were hand‐sorted from the sample in the field. Chemical extraction was performed simultaneously with hand‐sorting by the active agent in mustard, allyl isothiocyanate (AITC; mustard oil) (ISO, 2018). In the laboratory, 2 mL of AITC had been mixed into 40 mL of isopropanol, and in the field, just before the sampling, this stock solution was added in 20 L of water and mixed vigorously. The solution was applied to the bottom of the sampling pit and all emerging earthworms were collected with forceps. The amount of the solution used per pit varied from a couple of liters to more than 10 L depending on the pit size and infiltration capacity of the soil. The collection time was adjusted according to local conditions but lasted for at least 15–20 min.

Earthworms were washed in tap water and subsequently fixed in 4% formalin in the field or back at the laboratory. After a minimum fixation time of 24 h in formalin, the specimens were transferred to 70% ethanol. Before the identification, all specimens (intact individuals and parts of earthworms) were dried on a paper towel and weighed.

Adult and subadult earthworms (tubercula pubertatis present, but clitellum absent or not fully developed) were identified to species level using a stereo microscope and specialized taxonomic identification keys (Bouché, 1972; Csuzdi, 2007; Csuzdi & Zicsi, 2003; Graff, 1953; Herr & Bauchhenss, 1987; Krück, 2018; Sims & Gerard, 1999; Timm, 1999). Laboratory treatment of the material was divided between the partners so that samples from Mediterranean and Lusitanian zones were all analyzed in the University of Vigo (Spain), samples from Atlantic North and Continental zones at the Thünen Institute (Germany), samples from Atlantic Central and Pannonian zones at ILVO (Belgium), from Nemoral zone at the Institute of Agricultural and Environmental Sciences (Estonia), and from Boreal zone at Luke (Finland).

Here, data on species level are the sum of adults and subadult individuals. All specimens, also juveniles when possible, were assigned to their ecological groups (epigeic, endogeic, anecic), according to their pigmentation. Epi‐endogeic species with intermediate characteristics between epigeic and endogeic (Bouché, 1977) were also identified (epiendogeics feed on more nutritious food and are in that respect intermediate between primary and secondary decomposers). Earthworm data variables were defined as follows: (1) total abundance: all individuals including fragments with intact anterior end (“head”) that can be unequivocally counted (to avoid overestimation of population abundance); (2) total mass: all individuals and fragments (anterior and posterior ends and any middle parts); (3) identified species: those individuals and fragments with intact anterior end and clitellum or tubercula pubertatis that allowed taxonomical identification; (4) unidentified individuals: only fragments with intact anterior end when species identification was not possible. Earthworm total abundance and mass in samples were converted to per square meter. The number of species in a sample was used as such for the estimation of mean richness in a field.

### Soil properties and field management information

Simultaneously or very close in time with earthworm sampling, topsoil (0–0.25 m depth) composite sample was collected from the 1‐ha study area for characterization of site soil properties. Samples were collected with an auger at approximately 60 points, and the composite sample mass was at least 2 kg. A preliminary account of the soil data used in the present study is provided by Fernández‐Calviño et al. (2023). During the site visits, field management data were collected by interviewing farmers. For detailed descriptions of supplementary methods and additional information, see Fernández‐Calviño et al. (2020) and Sóto‐Gomez et al. (2020).

### Climate data

Air temperature and precipitation data for the study localities were collected from national databases. Air temperature data consisted of (1) the annual mean over the preceding 30‐year reference period, (2) mean of preceding 12‐month period, (3) mean of the lowest and highest temperatures during the preceding 12 months, (4) the number of days with temperatures under 0°C, and (5) the number of days with temperature over 30°C during the preceding 12 months. Precipitation data were collected for (1) mean annual precipitation over the preceding 30‐year reference period and (2) total precipitation over the preceding 12‐month period. Because of the strong correlation of different temperature and precipitation variables, the 30‐year annual mean temperature and precipitation data were used in the modeling of earthworm data.

### Statistical analyses

Earthworm metrics of a field (the response variables)—abundance (in individuals per square meter), mass (in grams per square meter), richness (number of species), proportion of ecological groups, and proportion of juveniles—were related to climate, soil properties, farming system, and field management (the potential explanatory variables). The main interest was first to disentangle the effects of farming system (conventional or organic) and pedo‐climatic conditions on communities. Mixed models were used in analyses to account for correlated data with three‐level hierarchy. There were nine pedo‐climatic zones, which were divided into altogether 82 localities, and which were represented by 188 fields in total (1–8 fields per locality). Response and potential explanatory variables are listed in Box 1.

BOX 1 **Earthworm response variables and potential explanatory variables in statistical modeling**

**Earthworm response variables:**

total abundance (in individuals per square meter), total mass (in grams per square meter), species richness (number of species), ecological group composition (in percentage of epigeic, endogeic, and anecic individuals), and age structure (in percentage of juvenile individuals)

**Potential explanatory variables:**

***Location*:**

pedo‐climatic zone (nine zones)

***Climate*:**

mean yearly temperature of 30 years (in degrees Celsius), mean yearly precipitation of 30 years (in millimeters)

***Soil*:**

texture (% of sand, silt, and clay), bulk density; mean of 0–10 and 10–25 cm depths (in grams per cubic centimeter), ${\mathrm{pH}}_{H_{2}O}$, soil organic matter content (in percent), element concentrations (P, N, Ca, K [in milligrams per kilogram]), and aggregate size (AMWD [in millimeters])

***Farming system*:**

system (organic, conventional)

***Field management*:**

tillage system^1^ (CT deep, CT shallow, RT deep, RT shallow, NT), fertilization (organic, mineral, organic + mineral, none), crop rotation system (short, long, monoculture), residue incorporation (yes, no), legumes in rotation (yes, no), and pesticide use (herbicides, combination, biocides, none)

^1^tillage system: CT, conventional tillage (plowing); NT, no‐till; RT, reduced tillage.

In the first step of the modeling, pedo‐climatic zone, farming system, and their interaction were used as explanatory (fixed) factors and locality as a random factor. In the second step, to find additional interpretative explanatory factors apart from pedo‐climatic‐zone, it was added as a random factor together with locality. The random factors were categorical variables in the mixed models to account for correlated (similar) response variable values from the same category.

Given the number of random factors in the models, all the response variables were non‐normally distributed (gamma, binomial, or negative binomial) and therefore modeled with generalized linear mixed models (Bolker et al., 2009), where the mean of a response variable (with the distribution‐specific nonlinear transformation) is modeled by a linear combination of fixed and random factors. Due to the nonlinear transformation, the model predictions for the mean of a response variable are generally nonlinear and especially curvilinear by the continuous explanatory variables. The modeling was performed using the GLIMMIX (the mixed models) and HPGENSELECT (searching of explanatory factors) procedures of SAS 9.4 software (SAS Institute Inc., 2018).

In the second step of the analysis, possible explanatory factors (including interactions and polynomial terms of continuous variables) were searched for all the response variables separately by the HPGENSELECT procedure with a stepwise model‐building method. The significance test for factor selection/dropping and Schwarz's Bayesian information criterion (BIC) were used for model selection. As the procedure is intended for the generalized linear models assuming independent data, the final mixed models were constructed by the GLIMMIX procedure to account for correlated data, accepting—in addition to farming system—the explanatory factors with significant (*p* < 0.05) and understandable effect in the model. In addition, some interesting factors were investigated individually. The competing mixed models with different explanatory factors, but the same random factors, were evaluated using the unexplained variation (the sum of random factor variances) and two information criteria (Schwarz's BIC and corrected Akaike information criterion for small sample [AIC_c_]) with the basic rule: the model with the smallest values is better. If the values were about the same for the competing models, the simplest model with the smallest number of parameters was selected.

The Tukey–Kramer method (Day & Quinn, 1989) with an overall significance level of 0.05 was used in pairwise comparisons of the model predicted class means by the explanatory factors. Additionally, a SAS macro was used for the display of results from pairwise comparisons (Piepho, 2012). The average model predictions were presented for the final models in the second step of the analyses, otherwise box‐and‐whisker plots of the data were used to describe the test results of the models. In the graphical display of the results, “ggplot2” package of R software was used (Wickham, 2016).

## RESULTS

### Variation in climatic and edaphic conditions

**FIGURE 2:**
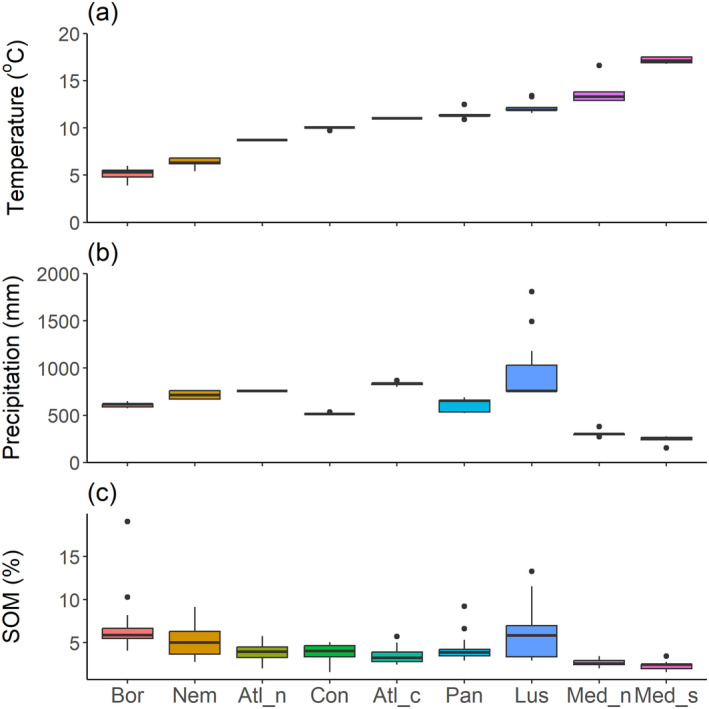
Box‐and‐whisker plots for long‐term climate and soil organic matter content in the nine European pedo‐climatic zones: (a) mean annual temperature over 30 years (in degrees Celsius), (b) mean annual precipitation over 30 years (in millimeters) and (c) Soil organic matter (SOM) content (in percent). Zones are ordered from north (left) to south (right): Bor, Boreal (Finland); Nem, Nemoral (Estonia); Atl_n, Atlantic North (Denmark); Con, Continental (Germany); Atl_c, Atlantic Central (Belgium); Pan, Pannonian (Hungary and Serbia); Lus, Lusitanian (Spain); Med_n, Mediterranean North (Spain); Med_s, Mediterranean South (Spain).

The climatic gradient from southern to northern zones was particularly consistent for temperature (Figure 2a). The latitudinal gradient of precipitation was less consistent due to regions close to the Atlantic Ocean receiving relatively much more rain (Figure 2b). Soil organic matter (SOM) content showed a generally increasing trend from south to north, with the highest values being measured at Boreal fields (Figure 2c). The soils were loams, with varying particle size distributions, except for one clay soil at Pannonian zone and the textural variation did not show any obvious geographical gradients (soil data available in Fernández‐Calviño et al., 2023).

### Variation in farming practices

**FIGURE 3:**
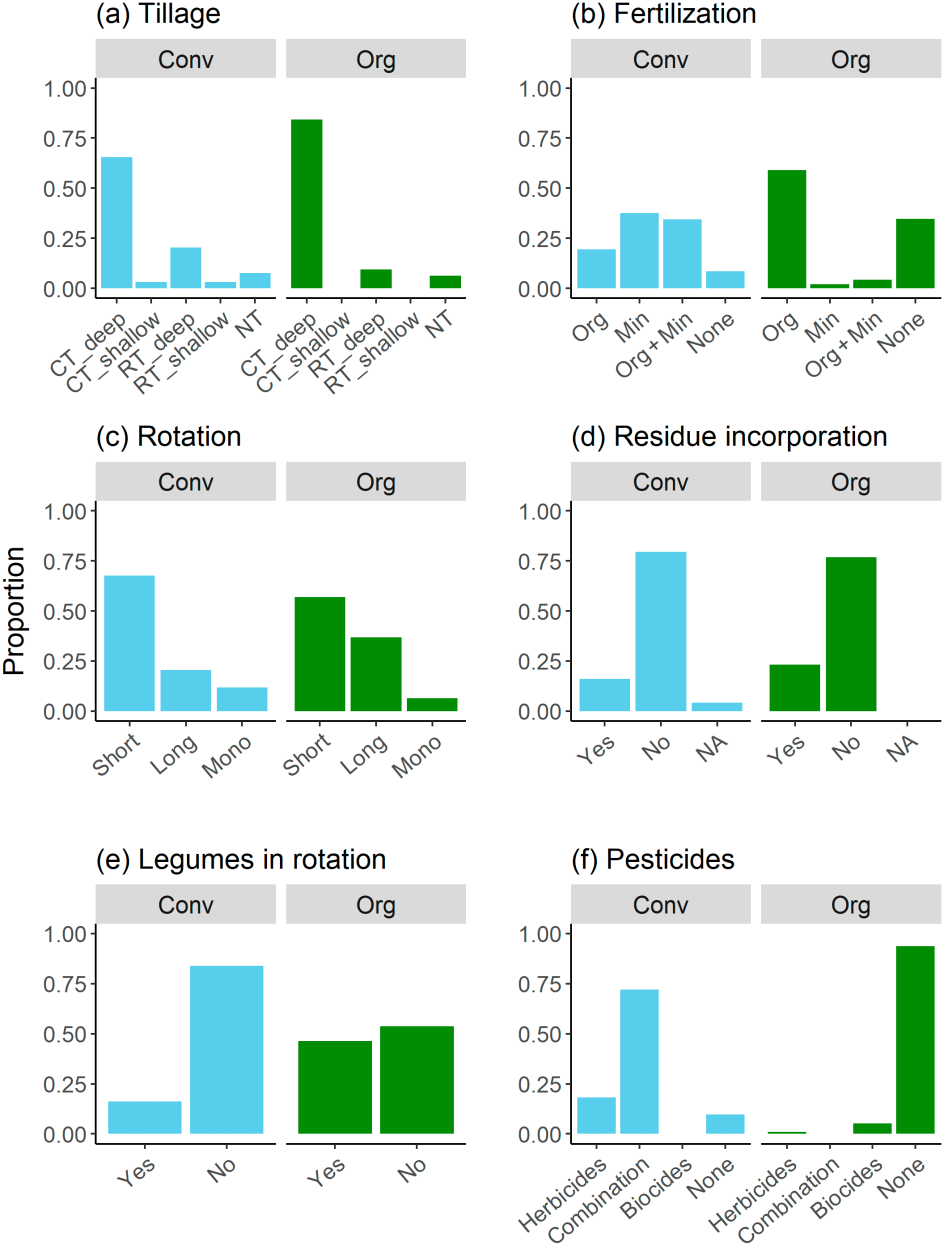
Distribution of management practices in organic and conventional systems in the combined data presented as the proportion of the fields (organic fields: *N* = 95, conventional fields: *N* = 93). (a–d) Conv, conventional farming; Org, organic farming; (a) CT, conventional moldboard tillage; RT, reduced tillage; NT, no‐till; (b) Org, organic fertilization; Min, mineral fertilization; None, no fertilization; (c) Mono, monospecific cultivation, no rotation.

Across all pedo‐climatic zones, organic farming was characterized by a higher frequency of conventional deep (moldboard) tillage while different reduced tillage options were somewhat more common under conventional farming (Figure 3a). Mineral fertilizers were used in conventional farming either alone or in combination with organic fertilizers (Figure 3b). Rotation lengths were highly similar in the two systems (Figure 3c) and the same held for residue incorporation, with residues usually not being incorporated (Figure 3d). In organic farming, legumes were included in rotation more often than in conventional systems (Figure 3e) and pesticides were usually not applied (Figure 3f). In multiple correspondence analysis ordination pesticide use was the factor that most clearly separated organic and conventional systems, with differences in fertilization and tillage also being influential (Appendix S1: Figure S1).

### Variation in earthworm abundance and mass

**FIGURE 4:**
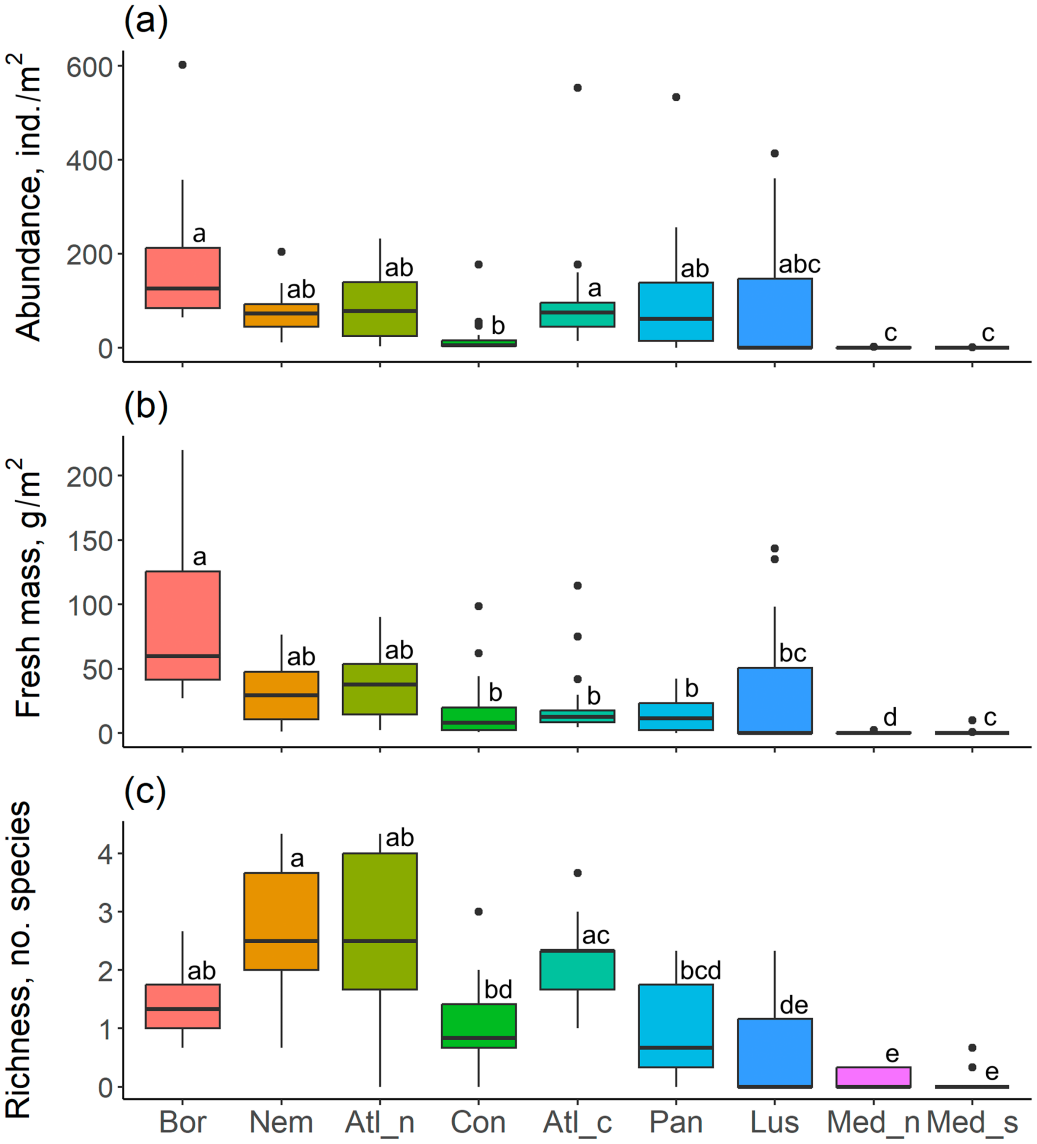
Box‐and‐whisker plots for zonal variation of earthworm community metrics: (a) earthworm total abundance (in individuals per square meter), (b) earthworm total mass (in grams per square meter), and (c) number of earthworm species. Pedo‐climatic zones denoted with the same letter were not statistically significantly different (*p* > 0.05) based on the mixed models using pedo‐climatic zone, farming system, and their interaction as explanatory factors. For zone abbreviations, see Figure 2 legend.

Pedo‐climatic zone significantly explained the geographical variation of both earthworm abundance and mass (Table 1). The highest mean earthworm abundance was observed at the Boreal zone and the lowest at the Mediterranean zones (Figure 4a). In the latitudes between them, the abundance was relatively similar, except for the low abundance in the Continental zone. In four zones—Boreal, Atlantic Central, Pannonian, and Lusitanian—there were individual fields with exceptionally high abundance. The geographical variation of mass followed the same general pattern, with the highest mean masses being recorded at the Boreal zone and the lowest at Mediterranean North and South (Figure 4b).

**TABLE 1:** Results of mixed model analyses using pedo‐climatic zone, farming system, and their interaction as the explanatory factors for earthworm total abundance, total mass, and richness in nine pedo‐climatic zones and two farming systems in Europe.

Effect	Abundance	Mass	Richness
	*p*‐value	*p*‐value	*p*‐value
Pedo‐climatic zone	**<0.0001**	**<0.0001**	**<0.0001**
Farming system	0.449	0.988	0.276
Pedo‐climatic zone × Farming system	0.628	0.523	0.382

*Note*: Significant results (*p* < 0.05) are given in bold.

**FIGURE 5:**
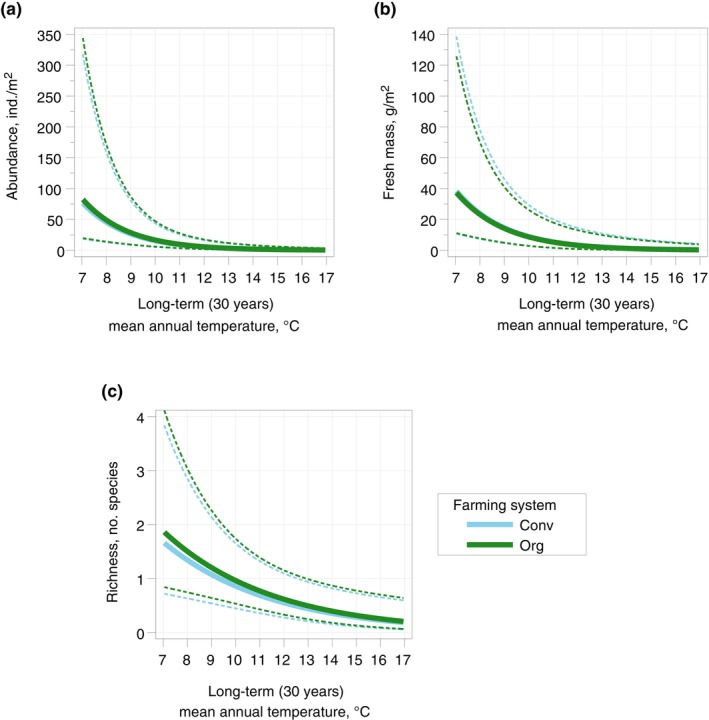
The average predictions by the mixed models using farming system and long‐term mean temperatures as the explanatory factors for (a) earthworm total abundance (farming system: *p* = 0.70; temperature: *p* = 0.0004), (b) earthworm total mass (farming system: *p* = 0.94; temperature: *p* = 0.002) and (c) earthworm richness (farming system: *p* = 0.53; temperature: *p* = 0.005). Dotted lines indicate 95% CI. Conv, conventional farming; Org, organic farming.

Of the environmental variables, the long‐term mean annual temperature showed a distinct and statistically significant negative relationship with both earthworm density and mass in the mixed models (Figure 5a,b, respectively).

**FIGURE 6:**
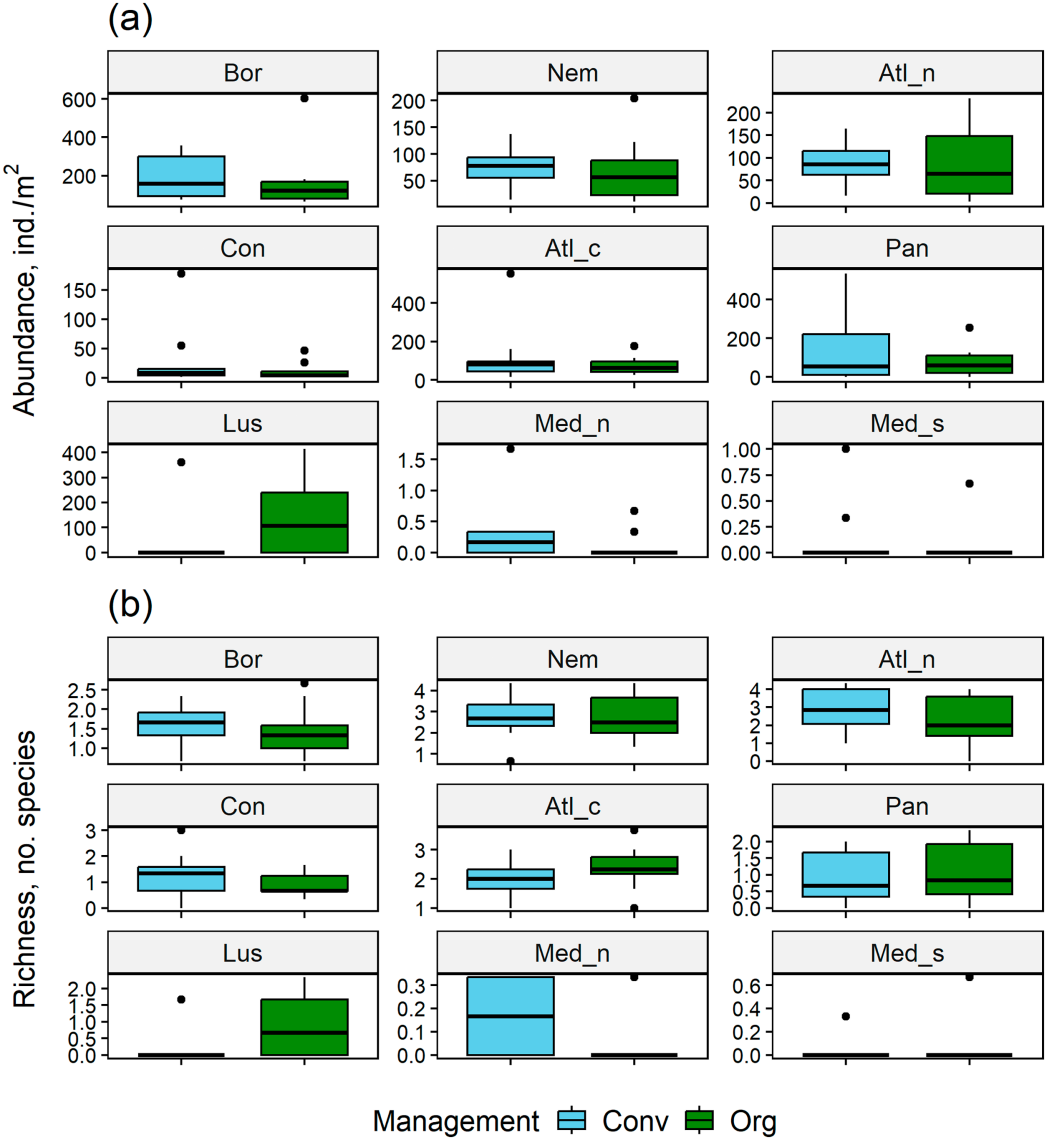
Box‐and‐whisker plots for earthworm abundance and species richness in relation to farming system: (a) earthworm total abundance and (b) earthworm species richness. None of the farming system differences within the regions were statistically significant (*p* > 0.05), based on the mixed models using pedo‐climatic zone, farming system, and their interaction as the explanatory factors. For zone abbreviations, see Figure 2 legend. Conv, conventional farming; Org, organic farming.

Farming systems (conventional vs. organic) did not differ statistically discernibly in earthworm abundance or mass (Table 1), and in none of the zones did the two systems differ from each other, as shown for earthworm abundance in Figure 6a (the result was the same for earthworm mass; data not shown). Notably, there was no interaction between the pedo‐climatic zone and the farming system for both earthworm abundance and mass (Table 1). For other field management practices and environmental factors, no statistically discernible associations with earthworm abundance and mass were detected. For different management practices, the results averaged across zones are summarized in Appendix S1: Figures S2 and S3.

### Variation in earthworm species richness

In total, 19 earthworm species were found at the nine pedo‐climatic zones (Table 2). Ten of the species recorded were endogeic, four anecic, three epigeic, and two epi‐endogeic. The three main groups (endogeic, anecic and epigeic) were present in all zones, except for epigeic species being entirely absent from the two Mediterranean zones. One species, the endogeic *Aporrectodea rosea*, was present in all nine zones. Other widely distributed endogeic species were *Allolobophora chlorotica* (seven out of nine zones) and *Aporrectodea caliginosa* (six zones). Of the anecic species, *Lumbricus terrestris* was found in five zones as was also for the epigeic species *Lumbricus rubellus*. The two epi‐endogeic species, *Octolasion tyrtaeum* (Boreal) and *Satchellius madeirensis* (Lusitanian), were both present in only one zone. Likewise, there were five endogeic and one anecic species found only in one zone.

**TABLE 2:** Presence of earthworm species (x) at the nine European pedo‐climatic zones.

Species	Group^a^	Pedo‐climatic zone^b^								
		Bor	Nem	Atln	Con	Atlc	Pan	Lus	Medn	Meds
*Eisenia fetida*	epig					x	x			
*Lumbricus castaneus*	epig	x	x			x				
*Lumbricus rubellus*	epig	x	x	x		x		x		
*Octolasion tyrtaeum*	epien	x								
*Satchellius madeirensis*	epien							x		
*Aporrectodea caliginosa*	endo	x	x	x	x	x	x			
*Allolobophora chlorotica*	endo	x	x	x	x	x	x	x		
*Aporrectodea georgii*	endo						x			
*Aporrectodea limicola*	endo					x				
*Aporrectodea rosea*	endo	x	x	x	x	x	x	x	x	x
*Microscolex dubius*	endo								x	
*Microscolex phosphoreus*	endo							x		
*Octolasion cyaneum*	endo		x		x	x		x		
*Octolasion lacteum*	endo					x	x			
*Proctodrilus antipai*	endo						x			
*Aporrectodea longa*	anec		x	x		x	x	x		
*Aporrectodea trapezoides*	anec						x	x		x
*Lumbricus friendi*	anec							x		
*Lumbricus terrestris*	anec	x	x	x	x	x				

*Note*: The species are divided into four ecological groups.

^a^ Ecological groups: anec, anecic; endo, endogeic; epiend, epiendogiec; epig, epigeic.

^b^ Pedo‐climatic zones: Atlc, Atlantic Central (Belgium); Atln, Atlantic North (Denmark); Con, Continental (Germany); Bor, Boreal (Finland); Lus, Lusitanian (Spain); Medn, Mediterranean North (Spain); Meds, Mediterranean South (Spain); Nem, Nemoral (Estonia); Pan, Pannonian (Hungary and Serbia).

The pedo‐climatic zone significantly explained the variation of richness within the fields (Table 1). The latitudinal pattern, where the maximum mean value remained close to two species, differed somewhat from those of earthworm abundance and mass, richness being highest at Nemoral and Atlantic North zones (Figure 4c). From there, it declined toward north and south, and was at the lowest in the southernmost sites (Figure 4c, Table 2). The variation of richness was also significantly negatively related to the long‐term mean yearly temperature (Figure 5c).

Earthworm species richness did not differ significantly between the two farming systems, and there was no interaction between zone and farming system (Table 1 and Figure 6b). The other environmental and management factors did not have statistically discernible effects on richness either. The result for management differences averaged across zones is summarized in Appendix S1: Figure S4.

### Variation of ecological groups and age structure

One management effect on ecological group composition was detected: in both farming systems, the proportion of endogeic species in the community was significantly larger when residues were incorporated in the soil (Appendix S1: Figure S5a). In the case of the age structure, the proportion of juveniles was significantly higher under organic farming across the rotations (Appendix S1: Figure S5b).

## DISCUSSION

### Environmental effects on earthworm abundance

In our survey, pedo‐climatic zone was overridingly important in explaining the observed variation of earthworm communities (research question (1)). The finding is parallel with two earlier research syntheses which have covered all land uses. In the global study of Phillips et al. (2019), precipitation and temperature were important drivers of earthworm abundance and local richness (alpha diversity), with European earthworm “hotspots” located at temperate Atlantic regions. In an earlier European study synthesis, the model estimates of earthworm abundance were particularly low in southern Mediterranean regions—as in the present study—and at its highest in central and northern Atlantic regions (Rutgers et al., 2016). The latter study did not cover the northern regions of Europe, and hence, the present survey complements it further with evidence for relatively high earthworm abundance and masses at Boreal and Nemoral zones.

The anatomy and physiology of earthworms correspond with their oligochaete relatives in ancestral freshwater sediments (Turner, 2002), signifying their strong dependence on sufficient soil moisture and the challenges set to them by temperature extremes in terrestrial environments (Singh et al., 2019). While lacking morphological structures that defend against desiccation, earthworms are both behaviorally and physiologically adapted to the variation in soil moisture and temperature. During drought and frost, endogeic species can burrow into deeper soil layers, dig an aestivation chamber, and enter quiescence while anecic species can descend to the lower parts of their deep burrows to avoid unfavorable conditions (Edwards & Arancon, 2022; Singh et al., 2019). Earthworms have also metabolic adaptations for survival under drought (Bayley et al., 2010; Holmstrup et al., 2016) and frost (Holmstrup, 2002), which can be particularly important for epigeic species unable to dig deeper during unfavorable periods.

The general conception is that high soil temperatures and drought pose a harder challenge for earthworm survival and population growth than frost or waterlogging of soils (Edwards & Arancon, 2022; Nordström & Rundgren, 1974). Despite the insufficient knowledge of drought impacts on earthworms, the available evidence suggests negative effects on earthworm activity and diversity (Singh et al., 2019). This is supported by our finding of highest earthworm abundance at the Boreal study sites where severe droughts are less common than in the more southern parts of Europe and where frost, on the other hand, typically lasts for 4–5 months and may be more than 0.5 m deep during cold winters in some of the study sites. It is possible that in the Boreal zone earthworms have benefitted from the recent warming of winter months (Mikkonen et al., 2015), although in areas with associated decrease in snow cover, the probability of frozen soil conditions may have increased at the same time (Venäläinen et al., 2001). While summer droughts occur to some extent in all of our study regions, they are most severe in the Mediterranean zones where the lowest earthworm abundance and masses were measured. In these regions, drought severity and intensity have also increased during the last decades (Vicente‐Serrano et al., 2014), which is likely posing earthworm survival at risk.

Long‐term data on global warming impacts on earthworms are still largely lacking, but Barnes et al. (2023) recently suggested that it could be one factor behind earthworm population declines in the southern parts of England. Such population declines could be the result of lowered fecundity, and mortality can also occur during severe and prolonged droughts, even though local populations of common endogeic species such as *A. rosea* and *A. caliginosa* can be genetically adapted to dry and hot conditions (Edwards & Arancon, 2022). Drought‐related declines in earthworm diversity and abundance will have cascading effects on soil processes, as documented by da Silva et al. (2020) in a mesocosm study where the decline of earthworm functional diversity in three European pedo‐climatic zones resulted in lower litter mass loss in all land uses studied.

To evaluate the climate impacts on earthworm communities more accurately, future studies should preferably use soil temperature data rather than the air temperature proxy used by us, although the utility of air temperature has been recognized in the study of soil warming (Dorau et al., 2022). It is also noteworthy that the interaction between soil temperature and moisture affects earthworms, for instance, their growth rate (Edwards & Arancon, 2022; Eriksen‐Hamel & Whalen, 2006). Further, as our study was based on sampling at a single time instance, the results could have been affected by the variation in local weather conditions during the weeks before and at the time of the sampling. We consider, however, that temporal adjustment of sampling time according to local weather conditions sufficiently guaranteed that variation in sampling efficiency between the sites did not seriously bias the results. The fact that a severe drought forced us to postpone earthworm sampling at two pedo‐climatic zones for a year is one sign of the ongoing rapid warming trend in European climate.

### Environmental effects on earthworm species richness

The richness within each field (alpha diversity) remained below three, even in zones with the highest number of species (research question (1)). Also, here our findings are in line with those of Phillips et al. (2019) who estimated at a global scale an average richness of two species in fields managed for herbaceous production. In the earlier research synthesis, European estimates of earthworm alpha diversity were particularly high in northern and central Atlantic regions of the continent (Rutgers et al., 2016). Of the other regions covered by our survey, richness estimates of that study appeared relatively high also for central Europe and the Baltic region, while the south and north Mediterranean and Boreal regions have been reported to be more species poor (Phillips et al., 2019). Our results agree with these earlier established general patterns, but also show that richness can be high up to the Nemoral zone (the northern parts of Baltic region) and that Boreal fields are not as species poor as the Mediterranean ones. In northern Europe, the low species richness relates to the limited dispersal of earthworms after the last glaciation in Europe (Terhivuo, 1988). The impact of glaciation can still bear also on within‐country patterns in more southern, glaciation‐affected regions of Europe (Mathieu & Davies, 2014).

The great majority of the species encountered in the study were endogeic species. Their general dominance in arable soils relates importantly to the tolerance of mechanical soil disturbance (Edwards & Arancon, 2022). Post‐harvest burial of crop residues by tillage does not impede the subsurface‐feeding endogeics to the same extent as epigeic and anecic species (Briones & Schmidt, 2017), and endogeic populations such as those of *A. caliginosa* can recover remarkably quickly even when tillage has injured a notable proportion of the population (Boström, 1995). The ability of endogeics to withstand changes in soil moisture and temperature conditions further promotes their populations in agricultural soils where physical conditions can change rapidly, particularly in tilled soils. Notably, epigeic species, which are most directly affected by extremes of air temperatures (Briones et al., 2009; Eggleton et al., 2009), were not encountered in any of the warmest and most severely drought‐affected Mediterranean sites.

### Farming system and field management effects on earthworm community metrics

With respect to our research question (2), no significant differences in earthworm community metrics emerged between the organic and conventional farming systems. The result is somewhat unexpected, considering that earlier experimental studies (e.g., Birkhofer et al., 2008) and research syntheses (Bengtsson et al., 2005; Bertrand et al., 2015; Christel et al., 2021) have reported beneficial effects of organic farming on earthworms. However, it has also been noticed earlier that the benefits of organic farming for belowground biota may be considerably smaller than for aboveground biodiversity (Bengtsson et al., 2005; Flohre et al., 2011). In relation to this, one earlier Europe‐wide study did not find any consistent differences in earthworm richness or abundance between conventional and organic farming (Schneider et al., 2014). In that study, the great variation in the farm types included, and their nonuniform selection from different regions likely affected the result.

Our study focused on the cultivation of a particular cereal, but in our case, too, the within‐farming systems variation of management with multifactorial and nondichotomic differences between organic and conventional farming may have contributed to the similarity of their earthworm communities. This finding leads to our research question (3) on the importance of management effects within the farming systems. The application of organic soil amendments in organic farming is highly favorable for earthworms (Birkhofer et al., 2008; Curry, 2004), but in our dataset, organic manures were also used in conventional farming, either alone or combined with chemical fertilizers. Likewise, while leguminous plants, typical for organic rotations, are beneficial for earthworms (Schmidt et al., 2003), they were often included in conventional farming, too, with 23 organic versus 15 conventional fields having legumes in rotation. Rotation lengths did not vary greatly between organic and conventional fields, and residue management, a potential determinant of earthworm abundance and diversity (Melman et al., 2019), was also notably similar in the two systems. The importance of the latter for earthworm community structure became, however, evident as the community proportion of endogeics was higher under residue incorporation. This is likely due to the relative benefits of the practice for subsurface‐feeding species.

When Hagner et al. (2023) recently compared soil faunal communities in organically and conventionally farmed soils, they noticed that high earthworm abundance and mass were typical for dairy farming irrespective of whether the management was organic or conventional. The only instance of a clear difference in earthworm abundance between conventional and organic cereal farming systems was between an organic system (with higher earthworm abundance) compared with a conventional system where no organic amendments or lay phases in rotation were used (Hagner et al., 2023). In our study, similar but unaccounted variation due to farm type may have contributed to the lack of farming system differences.

A clear‐cut difference between organic and conventional farming was the more common usage of pesticides in conventional farming. Widely applied pesticides often have negative effects on earthworms in laboratory tests and are potentially harmful to earthworms also in field conditions (Beaumelle et al., 2023; Gunstone et al., 2021; Pelosi et al., 2014). High actual risks may, however, not appear under cereal field crop cultivation, where necessary and maximal allowed application rates are often benign compared with, for instance, those used for root crops or in orchards. Our data on pesticide usage was robust for refined statistical analyses, but it is reasonable to assume that herbicides constituted a large proportion of the usage in conventional wheat fields. Compared with insecticides and fungicides, they are known to be less harmful to earthworms (Pelosi et al., 2014). Therefore, pesticide application may not have been a highly important factor influencing earthworms in our study (research question (3)).

Applied tillage method is an example of management practice with well‐established and often large impacts on earthworm abundance and community composition (Briones & Schmidt, 2017). In our dataset, reduced tillage and no‐till were more common under conventional farming than in organic farming where the usage of inversion tillage with moldboard plow is often required also for weed control. This difference in tillage regime between the farming systems was a likely factor offsetting other potential benefits of organic farming (research question (3)).

### Farming systems and vulnerability of earthworms to climate change

A topical challenge in the management of European arable soils is how to increase their resilience to severe droughts, which presently constitute a continent‐wide threat to field crop production (Brás et al., 2021). Adoption of organic and regenerative farming could alleviate some of the drought‐related problems by, for instance, increasing the soil water holding capacity through increased SOM content (Droste et al., 2020). This relates closely to the future of arable soil animal communities including earthworms under the changing climate and soil moisture conditions (Hiltpold et al., 2017; Singh et al., 2019). Earlier Meyer et al. (2021), for instance, noticed that organic farming buffered the negative effects of drought on soil microarthropods, possibly due to higher SOM content and water holding capacity and enhanced soil structure.

The measurements in our study sites, however, did not provide indications that organic farming could alleviate drought‐related challenges for earthworms through more favorable soil conditions (research question (2)). Our previous analyses (Fernández‐Calviño et al., 2023) namely showed that only in the Lusitanian zone the mean SOM was significantly higher in organic than in conventionally farmed fields (7.2% vs. 4.1%). In other zones, no differences between these two farming systems were detected or SOM was significantly lower in organically managed soil (2.4% vs. 3.0% in Mediterranean North). The moisture readings during the soil sampling did in turn not indicate higher soil moisture in organically managed fields (Fernández‐Calviño et al., 2023). It is, however, possible that uncontrolled or unaccounted variation in the environment and management weakened the statistical comparison of the farming systems. One potentially important factor not covered by this study is the habitat complexity of the landscape (Flohre et al., 2011).

The high importance of temperature and precipitation on the variation of earthworm communities has signaled that climate change is likely to have major impacts on earthworm abundance and diversity (Phillips et al., 2019). Our findings of low earthworm community metrics in the warmest and driest zones of the study together with the general warming and increasingly common droughts in many regions of Europe suggest potential future deterioration of living conditions of earthworms. A recent modeling study also indicated that under future climate scenarios, the geographical ranges of most earthworm species in Europe will change and that some populations will face a decrease (Zeiss et al., 2024). The risks may be elevated by the increase of rapidly onsetting flash droughts (Christian et al., 2023; Yuan et al., 2023) where behavioral and physiological adaptation of earthworms can be compromised. In drought‐affected agricultural soils, the risk of earthworm decline is a matter of concern considering the contribution of earthworms on soil fertility, particularly in low‐input systems (van Groenigen et al., 2014) and their global contribution to global grain yield (Fonte et al., 2023). With no indication of pedo‐climatic zone and farming system interaction, our results do not provide indications that organic management alone could alleviate the trials caused by the warming climate.

Due to their importance in soil processes and sensitivity to land management practices, earthworms are often considered as potential biological indicators of arable soil quality (EEA, 2023; Griffiths et al., 2016). This view has, however, been challenged due to the typically large within‐field variation of earthworm numbers, often not obviously related to the variation of other indicators of soil quality, and because of the prominent importance of uncontrolled climatic regulation of earthworm abundance (Hodson et al., 2021). The present results underline that the usage of earthworms as indicators requires consideration of the notable zonal variation in earthworm communities and the possible climate‐related temporal trends not directly related to land management.

## CONFLICT OF INTEREST STATEMENT

The authors declare no conflicts of interest.

## Supporting information


Appendix S1.

## Data Availability

Data (Nuutinen et al., 2024) are available in Zenodo at https://doi.org/10.5281/zenodo.13822786.
