# Supplementary material for: Wheat field earthworms under divergent farming systems across a European climate gradient
Source: Ecol Appl. 2024 Nov 25;35(1):e3066. doi: 10.1002/eap.3066 (PMC11734576; doi:10.1002/eap.3066)
Supplement: Supplementary file 1 — Appendix S1. [file EAP-35-e3066-s001.pdf]

Appendix S1

Wheat field earthworms under divergent farming systems across a European climate gradient

Visa Nuutinen, Maria J.I. Briones, Stefan Schrader, Igor Dekemati, Nikola Grujić, Juha Hyvönen, Mari Ivask, Simon Bo Lassen, Eva Lloret, Irene Ollio, Paula Pérez-Rodríguez, Barbara Simon, Merit Sutri, Nancy de Sutter, Kristian K. Brandt, Krista Peltoniemi, Merrit Shanskiy, Lieven Waeyenberge, Silvia Martínez-Martínez, David Fernández-Calviño

Journal: Ecological Applications

Table S1. Number of sampled fields and time of sampling at the pedo-climatical zones. Conv=Conventional farming; Org=Organic farming.

Zone (abbreviation)	Country	Number of fields		Time of sampling	
		Conv	Org	Year	Month(s)
Boreal (Bor)	Finland	10	10	2019	Sep
Nemoral (Nem)	Estonia	10	10	2019	Aug-Sep
Atlantic north (Atl_n)	Denmark	10	10	2019	Aug
Continental (Con)	Germany	10	10	2019	Oct
Atlantic central (Atl_c)	Belgium	13	12	2019	Oct-Nov
Pannonian (Pan)	Hungary	10	10	2020	Oct-Nov
	Serbia			2020	Jul
Lusitanian (Lus)	Spain	10	13	2019	Jul
Mediterranean north (Med_n)	Spain	10	10	2020	Jan-Mar
Mediterranean south (Med_s)	Spain	10	10	2020	Jan-Mar

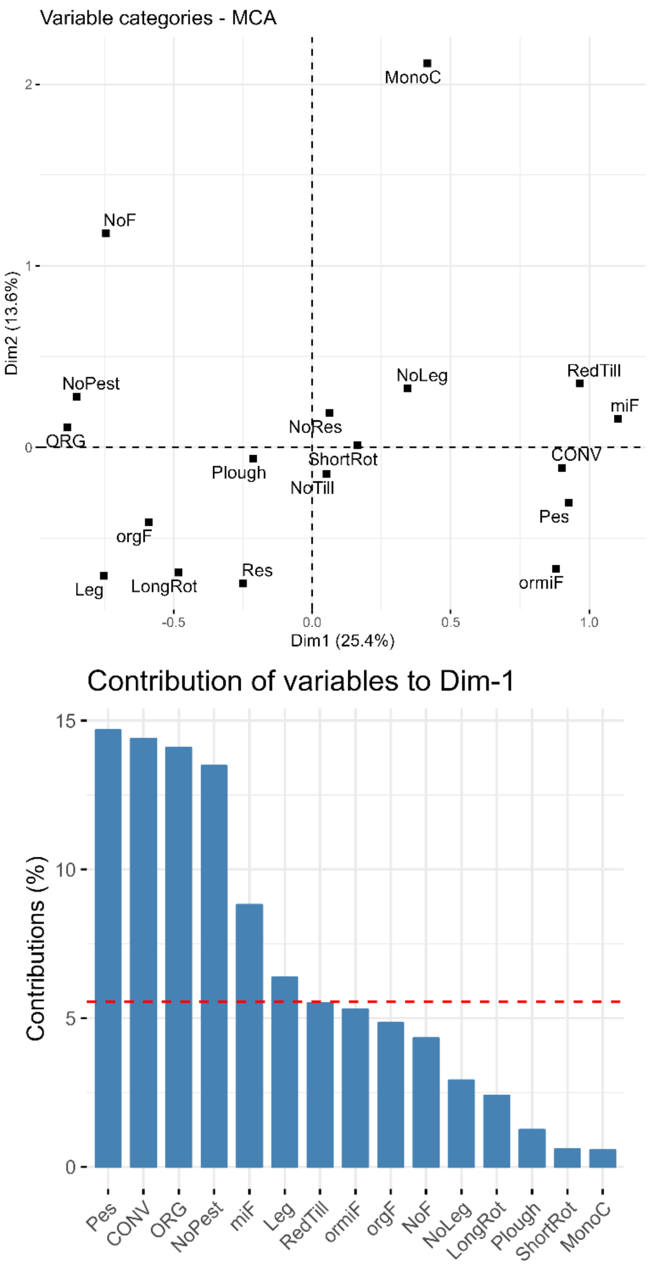

Figure S1. Multiple correspondence analysis (MCA) showing the relationships between management practices and farming systems. Results are presented in two-dimensional ordination plot (above) and as contribution of variables in dimension 1 separating farming systems (below). Abbreviations: CONV, conventional farming system; ORG, organic farming system; Plough; mouldboard plough; RedTill, reduced tillage; NoTill; no tillage; MonoC, wheat monoculture; ShortRot, rotation cycle for 2-3 years,; LongRot; rotation cycle of 4 or more years; NoF, no fertilization; miF, mineral fertilization; orgF, organic fertilization; ormiF, both organic and mineral fertilization; NoLeg, legumes not used in rotation; Leg, legumes used in rotation; NoRes, no crop residues left on the field; Res, crop residues left on the field; NoPest, pesticides not used; Pes, pesticides used. The red dashed line indicates the expected average value if the contributions of variables were uniform. Source: Peltoniemi et al. 2024.

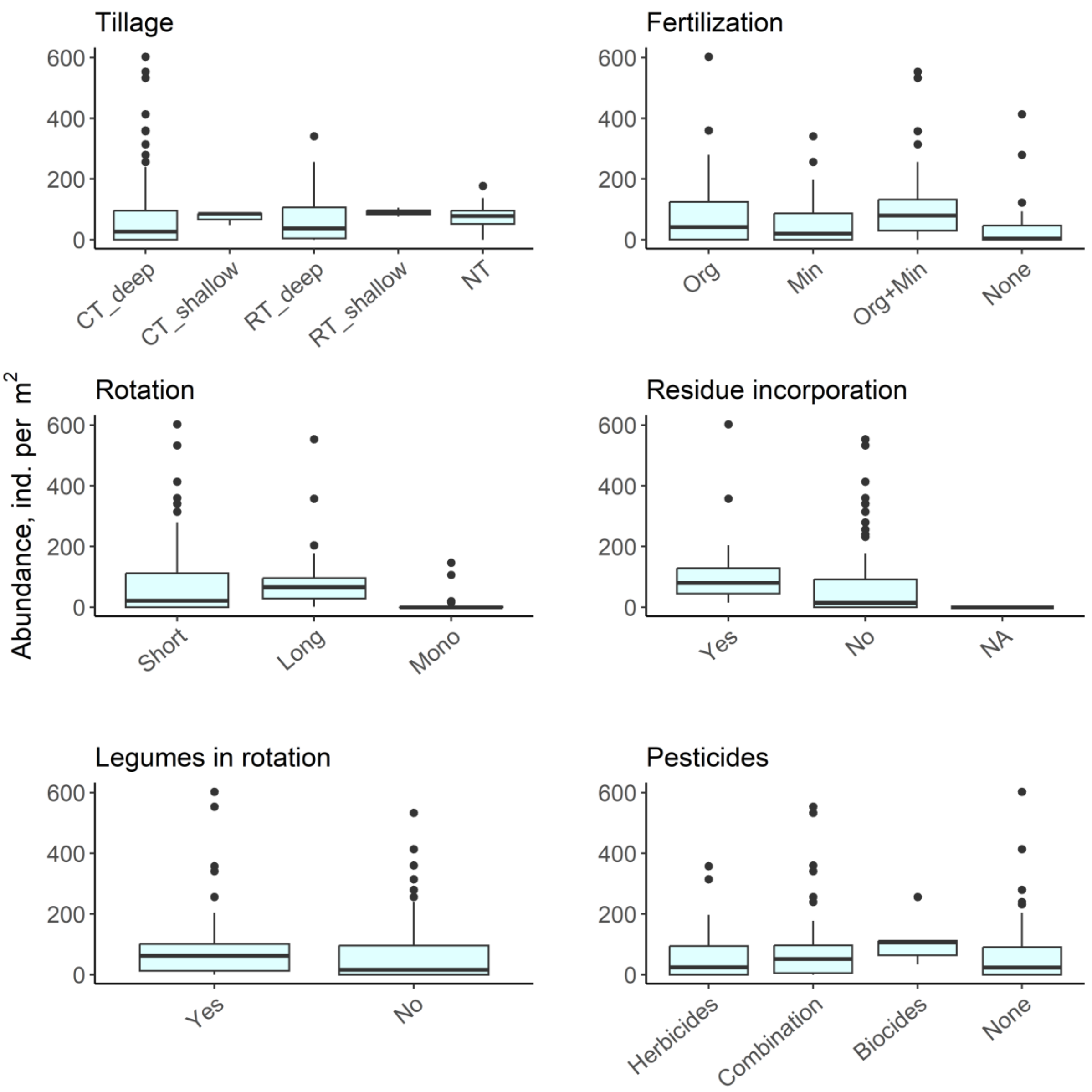

Figure S2. Box-and-whisker plots for earthworm total abundance in classes of main explanatory categorical variables. Data is combined over pedo-climatic zones and farming systems. None of the class differences were statistically significant ($p>0.05$) when the categorical variable was added in the mixed model (Table 2).

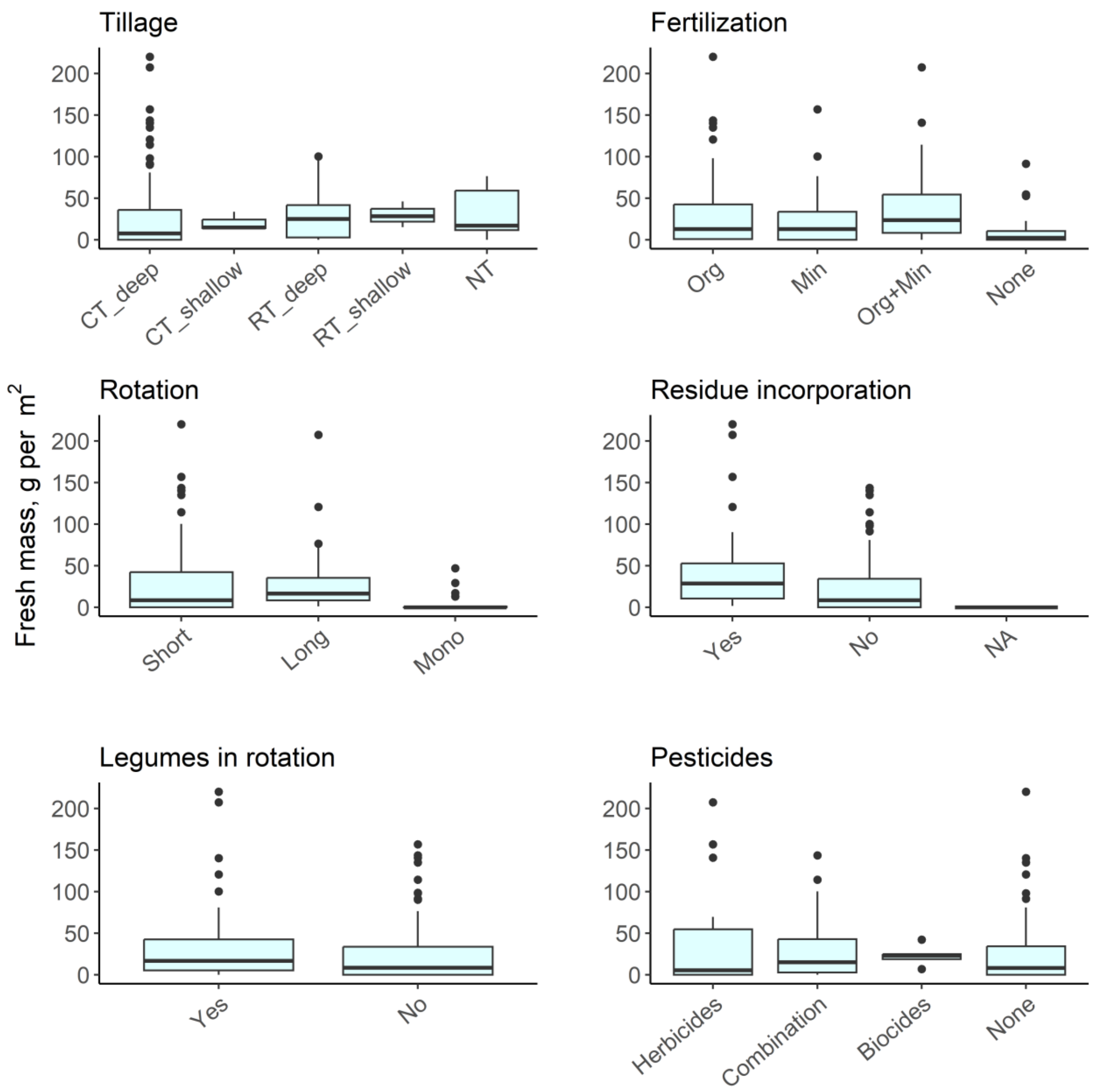

Figure S3. Box-and-whisker plots for earthworm total mass in classes of main explanatory categorical variables. Data is combined over pedo-climatic zones and farming systems. None of the class differences were statistically significant (p>0.05) when categorical variable was added in the mixed model (Table 2).

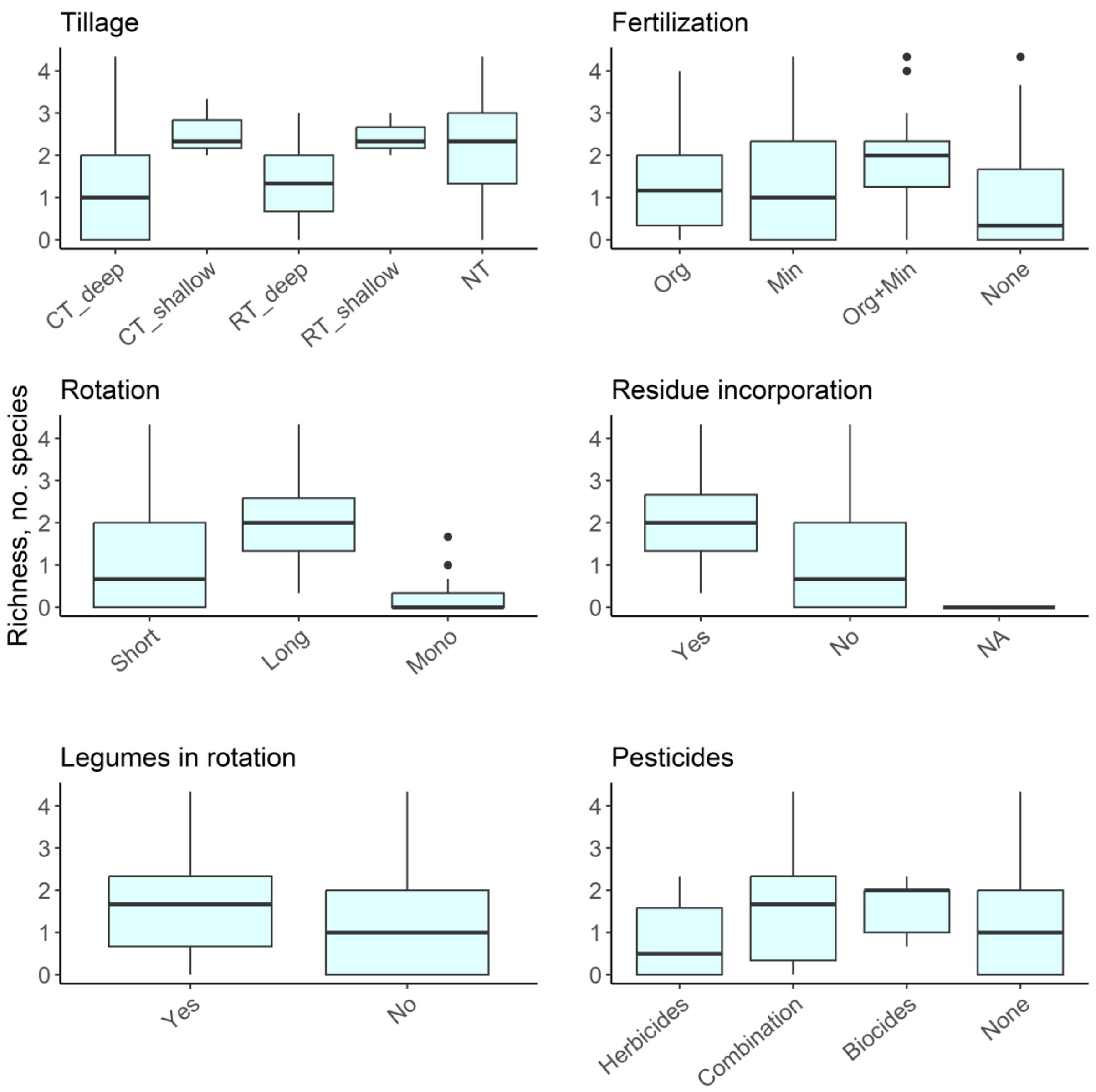

Figure S4. Box-and-whisker plots for earthworm species richness in classes of main explanatory categorical variables. Data is combined over pedo-climatic zones and farming systems. None of the class differences were statistically significant ($p>0.05$) when categorical variable was added in the mixed model (Table 2).

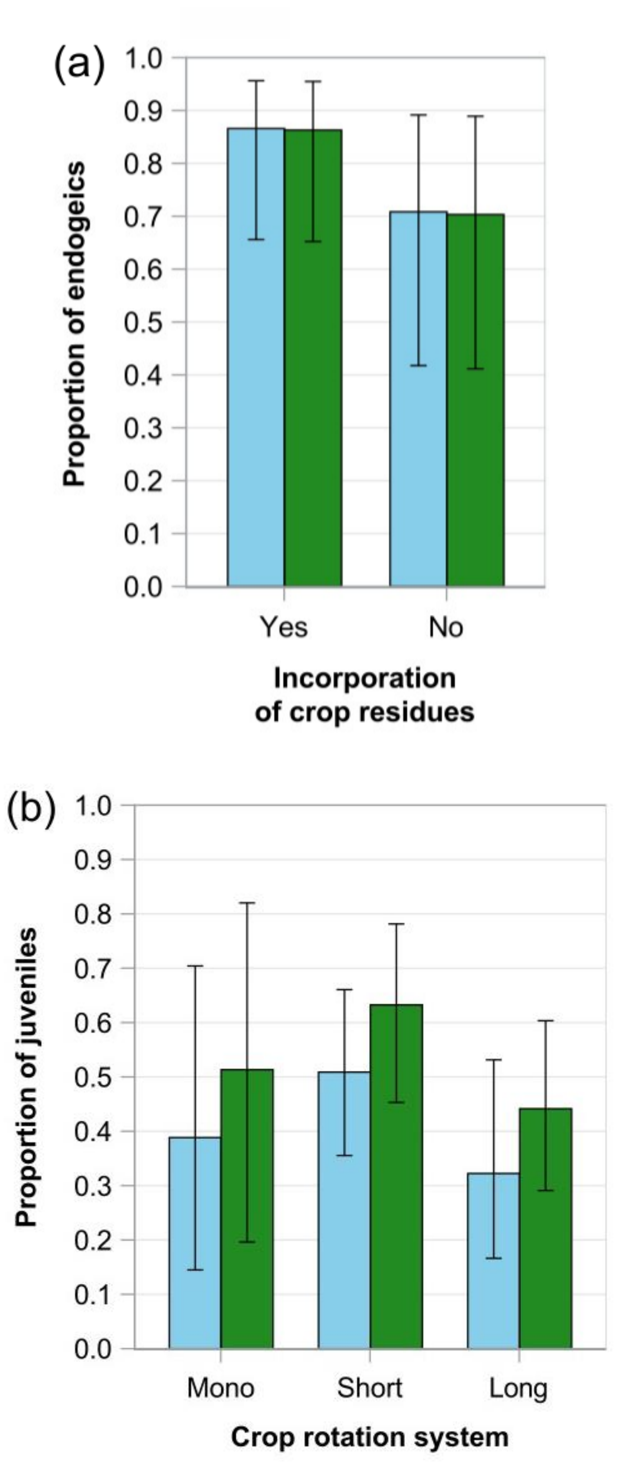

Figure S5. The average predictions by the mixed models for (a) proportion of endogeic earthworms using farming system ($p=0.83$) and crop residue incorporation ($p<0.0001$) as the explanatory factors, and (b) proportion of juvenile earthworms using farming system ($p=0.012$) and and crop rotation ($p=0.046$) as the explanatory factors. Blue bars: conventional farming; green bars: organic farming. Vertical lines denote 95% confidence interval.

References

Peltoniemi, K., S. Velmala, E. Lloret, I. Ollio, J. Hyvönen, E. Liski, K.K. Brandt, C. Campillo-Cora, H. Fritze, S. Iivonen, S.B. Lassen, K. Loit, S. Martínez-Martínez, T. Pennanen, M. Põldmets, S. Schrader, M. Shanskiy, R. Zornoza, L. Waeyenberge, and D. Fernández Calviño 2024. "Soil and climatic characteristics and farming system shape fungal communities in European wheat fields." Agriculture, Ecosystems & Environment 370: 109035.
